# A life span perspective on competencies for a healthy, physically active lifestyle: Findings of a data pooling initiative with over 7000 individuals

**DOI:** 10.1002/ejsc.12100

**Published:** 2024-04-16

**Authors:** Johannes Carl, Simon Blaschke, Gorden Sudeck, Julia Schmid, Katharina Eckert, Wolfgang Geidl, Johannes Jaunig, Maximilian Köppel, Joachim Wiskemann, Anna‐Maria Liphardt, Klaus Pfeifer

**Affiliations:** ^1^ Institute for Physical Activity and Nutrition, Deakin University Geelong Australia; ^2^ Department of Sport Science and Sport Friedrich‐Alexander‐Universität Erlangen‐Nürnberg Erlangen Germany; ^3^ School of Medicine and Health Technical University Munich Munich Germany; ^4^ Institute of Sports Science University of Tübingen Tübingen Germany; ^5^ Institute of Sport Science University of Bern Bern Switzerland; ^6^ Health Management & Public Health IST‐University of Applied Sciences Düsseldorf Germany; ^7^ Institute of Human Movement Science, Sport and Health University of Graz Graz Austria; ^8^ Heidelberg University Hospital and NCT Heidelberg a partnership between DKFZ and University Medical Center Heidelberg Heidelberg Germany; ^9^ Medizinische Klinik 3 – Rheumatologie & Immunologie Deutsches Zentrum Immuntherapie Friedrich‐Alexander‐Universität Erlangen‐Nürnberg & Universitätsklinikum Erlangen Erlangen Germany

**Keywords:** age, competence, exercise, health, physical activity

## Abstract

Individuals are recommended to lead active lifestyles throughout the life course. The model of physical activity‐related health competence (PAHCO) adopts a competence approach by integrating physical, cognitive, and motivational determinants for health‐enhancing PA (movement competence, control competence, self‐regulation competence). Drawing on a comprehensive dataset pooling, the goal of the present study was to model the idiosyncratic courses of 10 PAHCO indicators over the life span. We identified studies that empirically operationalized PAHCO, combining data of 7134 individuals (age range: 15–97 years; 61% female) from 18 different populations (prevention and rehabilitation sectors). We applied a stepwise multilevel analysis approach with disjunct sub‐samples (*n* = 48) to examine linear and quadratic associations between age and PAHCO. Indicators of movement competence (i.e., manageability of endurance, strength, and balance demands; task‐specific self‐efficacy) congruently showed negative associations with age (0.054 ≤ $R_{\text{marg}}^{2}$ ≤ 0.211). However, parameters of control competence remained stable across the life span (−0.066 ≤ *β* ≤ 0.028). The three indicators of self‐regulation competence revealed an inconsistent relationship with age, though uncovering positive associations for self‐control (*β* = 0.106) and emotional attitude toward PA (*β* = 0.088). The associations of some indicators varied significantly across sub‐samples. The results suggest differential analyses for associations between PAHCO and age. While the physically determined PAHCO indicators (movement competence) probably decline across the life span, the ability to ensure regularity of PA (self‐regulation competence) or align PAs with an individual's health (control competence) appear to remain constant or improve with increasing age. The findings reinforce a de‐stigmatizing approach for PA promotion practices with considerable space for aligning activities with health also in the elderly.

## Highlights

- As hypothesized, movement competence appears to decline throughout the life span.
- The ability to align physical activities with an individual's health (control competence) appears to remain stable throughout the life span.
- Unexpectedly, an individual's ability to ensure regularity of physical activity (PA; self‐regulation competence) appears to improve throughout the life span and, therefore, conflicts with actual PA behavior.
- Age‐related changes in competencies for health‐enhancing PA systematically vary between samples (i.e., groups with a certain disease or working environment).

**Abbreviations**
ICC: intraclass correlation coefficients
MBD: manageability of balance demands
MED: manageability of endurance demands
MSD: manageability of strength demands
MSE: mean standard error
PA: physical activity
PAHCO: physical activity‐related health competence
REML: restricted maximum likelihood
RIM: random‐intercept model
RIOM: random‐intercept‐only model
RSM: random‐slopes model
WHO: World Health Organization

## 1 INTRODUCTION

Individuals face different challenges throughout their life course while undergoing dynamic physical alterations, social rearrangements, and changing psychological demands (Hutteman et al., 2014; Pinquart & Pfeiffer, 2020). Health aspects gain increasing importance across the life trajectory and strongly affect an individual's quality of life (Hansen & Blekesaune, 2022). In this context, physical activity (PA) can be awarded a crucial role in fostering holistic health and well‐being (Daskalopoulou et al., 2017; Warburton & Bredin, 2017). Accordingly, achieving a sufficient level of PA is of substantial public health interest at any age. The most important recommendations on PA provide a differentiated approach by suggesting volumes (e.g., durations and frequencies) and sometimes practical actions tailored for certain age segments (King et al., 2019; Rütten et al., 2016; World Health Organization, 2020). For instance, the World Health Organization (2020) asks adolescents to perform at least 60 min of moderate‐to‐vigorous PA per week. Adults should achieve at least 150 min of moderate‐to‐intense PA per week and include muscle‐strengthening activities at least two times per week (World Health Organization, 2020). Specifically, older adults and adults with chronic conditions are advised to additionally emphasize functional balance exercises at least three times per week (Rütten et al., 2016; World Health Organization, 2020). In general, there is actually no age group, in which PA is discouraged or not explicitly recommended. However, empirical studies suggested that PA levels are related to age in a non‐linear fashion, with an increasing reduction starting around the age of 50 years (Varma et al., 2017; Westerterp, 2018).

**FIGURE 1:**
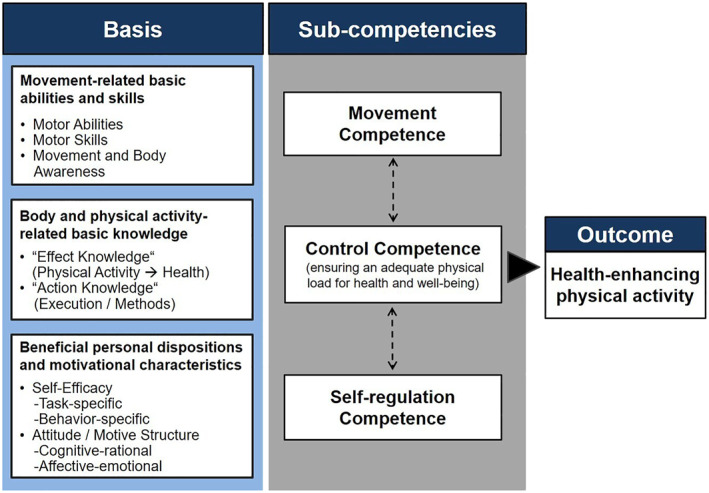
The physical activity‐related health competence model (Sudeck & Pfeifer, 2016).

Given the need to lead a physically active lifestyle coupled with changing actual PA levels throughout the life course, researchers and practitioners can benefit from comprehensively understanding the determinants of PA (Biddle et al., 2023; Rhodes et al., 2019). In this context, multidimensional approaches considering physical, cognitive, and motivational aspects of PA have gained substantial popularity, as these place the individual at the center of elaborate analyses and account for the complex nature of human health and behavior (Cairney et al., 2019; John et al., 2020; Wiklander et al., 2022). One of these comprehensive approaches is the physical activity‐related health competence (PAHCO) model which converges quantitative and qualitative determinants of health‐enhancing PA (Carl, Sudeck, & Pfeifer, 2020; Sudeck & Pfeifer, 2016). Drawing on the concept of “competence” originating in the educational sciences (Klieme et al., 2010), the PAHCO model assumes that a healthy, physically active lifestyle results from the integrative consideration of movement competence, self‐regulation competence, and control competence (Figure 1). *Movement competence* describes the individual ability (resulting from adequate body and movement awareness as well as motor abilities and skills) to adequately cope with direct movement‐related demands in exercise and everyday PA. *Self‐regulation competence* represents motivational and volitional aspects (e.g., including behavioral self‐efficacy, subjective attitudes, and motives) for ensuring a regular execution of PA. Finally, while the first two competencies are primarily compatible with the slogan “the more, the better”, *control competence* marks the qualitative aspect of health‐enhancing PA by aligning an individual's activities with holistic health (e.g., finding adequate physical loads of exercises or appropriate activities for psychological well‐being).

PAHCO has already been used in different contexts and target groups along the rehabilitation and prevention spectrum (Sudeck et al., 2022), encompassing, for instance, bariatric patients (Schmid et al., 2023), cancer survivors (Koeppel et al., 2021), persons with multiple sclerosis (Carl et al., 2021), office workers (Blaschke et al., 2023), apprentices (Grüne et al., 2022), and school children (Volk et al., 2021). Furthermore, PAHCO was empirically associated with the indicators of PA and health (Blaschke et al., 2021; Carl et al., 2021; Haible et al., 2020; Lindemann et al., 2023). In summary, no study has yet modeled courses of these holistic requirements for an active lifestyle over the life span or adopted a corresponding aging perspective. While an in‐depth understanding of PA levels with age (Varma et al., 2017; Westerterp, 2018) is crucial to describe the desired behavior outcome, an ontogenetic perspective on PAHCO has the potential to directly illuminate the modifiable determinants of PA in relation to the chronological age. To the best of our knowledge, the literature has not yet holistically described the person‐related requirements for (health‐enhancing) PA across the life span. Given the conceptually inherent plasticity of “competencies” (Weinert, 2001), such insights would be important for deriving age‐specific priorities for health‐enhancing PA, especially in the elderly. In terms of movement competence, several studies consistently underlined age‐related reductions in physical fitness as well as recess in instrumental activities of daily living and interoceptive qualities (Khalsa et al., 2009; Kimura et al., 2012; Liao & Chang, 2020; Mueller‐Schotte et al., 2020; Murphy et al., 2018; Tveter et al., 2014). Although studies have registered nuanced changes of psychological factors (Carstensen et al., 2006) and qualitative aspects of PA motivation (e.g., shifts in motives or in the type of motivation) with age (Louw et al., 2012; Molanorouzi et al., 2015; Steltenpohl et al., 2019), abstractions or specific conclusions for the course of self‐regulation competence and control competence (as specific constructs of the PA field) across the life span cannot be drawn. Against this backdrop, we formulated the following research questions for this study: How is age associated with competencies for healthy, physically active lifestyles? Does this association systematically vary between populations?

We followed two goals with the present study. First, our research team aimed to multi‐dimensionally model the relationship between age and PAHCO. Based on previous studies, we hypothesized that indicators of movement competence were significantly associated with age. However, for the indicators of control competence and self‐regulation competence we employed an exploratory approach by not specifying any hypotheses. Second, we analyzed whether age‐related associations can be assumed to be stable (invariant) or varying across target groups of PAHCO.

## 2 MATERIALS AND METHODS

### 2.1 Study design and participants

We conducted the analyses within the scope of an initiative aggregating datasets on PAHCO in German‐speaking countries. In summer 2021, we aimed to identify researchers that have encompassed any assessment of PAHCO in scientific studies via unstandardized web‐searches, contacts through conferences and published articles and members of the PAHCO network. We restricted the search to German‐speaking projects, as there have been no published validation studies with instruments in other languages than German. In winter and spring 2021/2022, the core team invited all researchers for discussing the opportunity to perform dataset pooling on PAHCO across different projects to enable analyses across populations and with stronger statistical power. After making organizational arrangements (e.g., data protection issues, contracts for data use and transfer) and defining common core items, the first author (JC) technically performed dataset pooling with SPSS v28 (IBM) by inverting and recoding overlapping variables, if necessary (for details, see Supplementary Table S1). The final pooled dataset comprised a total of 7233 individuals from 19 independent samples and 58 sub‐samples, respectively. Sub‐samples of a dataset were defined as groups *within* a study that shared a thematically relevant characteristic (focusing homogeneity *within* the sub‐samples) justifying disjunct treatment (characteristic maximizing heterogeneity *between* sub‐samples). More specifically, a disjunct treatment of category implied that the opportunity of a simultaneous assignment to two groups was strictly excluded at the study level and minimized at the pooled dataset level. As an indication of its relevance, the potential categorization information had to be already included within the original datasets. Furthermore, the assignment characteristic had to be thematically independent from the covariates (i.e., no simple assignment by gender or age groups). The most frequent reasons for the formation of sub‐samples were sub‐forms of diseases for rehabilitation studies and specializations of occupations for prevention studies. The final sample had a median age of 42 years and a mean age of 40.37 ± 16.78 years (age range: 15–97 years). The distribution across the life span was as follows: 3.2% of the participants were 5–17 years old, 34.8% were 18–29 years old, 15.1% were 30–44 years old, 33.6% were 45–59 years old, 12.1% were 60–74 years old, and 1.3% were 75 years or older. Across the primary studies, 61% of the participants self‐identified as female and 39% as male. Detailed information on the samples can be accessed in Table 1. All participants provided informed, written consent to study participation (see Table 1). Individuals under the age of 18 years additionally had to submit a written consent by their legal guardians. All included studies stood in line with the respective countries' ethical regulations at the time point of the conduction of the study.

**TABLE 1:** Overview of the different samples included.

Sample		Setting	Project responsibility (primary Publication)^a^	*N* ^b^	Mean age [range]	Gender^c^	Informed consent	Disjunct sub‐samples (entering this analysis)
1	Participants of a university sports program	Cross‐sectional survey within fitness‐ and health‐related programs of university sports at a university in Baden‐Württemberg, Germany	University of Tübingen (Sudeck & Pfeifer, 2016)	1374	26.59 ± 9.67 [range 17–75]	12.1% male, 87.9% female	X	Target group: (a) students (*n* = 1070), (b) employees (*n* = 108), (c) guests (*n* = 174), (d) undetermined (*n* = 22)
2	In‐patient rehabilitants	Written interview at the beginning of a medical rehabilitation program in 10 rehabilitation facilities		1028	53.75 ± 9.24 [range 19–88]	56.0% male, 44.0% female	X	Indication group: (a) orthopedics/back (*n* = 340), (b) metabolic (*n* = 230), (c) cardiology (*n* = 194), (d) oncology (*n* = 157), (e) orthopedics/joint (*n* = 107)
3	Persons with diabetes	Cross‐sectional online survey for disease‐specific associations, self‐help groups, and social media groups	FAU Erlangen‐Nürnberg (not published)	194	43.32 ± 13.91 [range 18–77]	27.1% male, 72.9% female	X	Type of diabetes: (a) diabetes type 1 (*n* = 105), (b) diabetes type 2 (*n* = 72)
4	Vocational students in automotive mechatronics	Vocational students of Bavarian institutions, pre‐intervention needs assessment of a scientific project (PArC‐AVE)	FAU Erlangen‐Nürnberg (Carl, Sudeck, Geidl, et al., 2020)	496	18.19 ± 2.20 [range 15–31]	82.4% male, 17.6% female	X	Automotive apprentices: (a) mechatronics (*n* = 307), (b) manufacturing mechanics (*n* = 104), (c) vehicle mechanics (*n* = 65)
5	Vocational students of nursing care	Vocational students of Bavarian institutions, pre‐interventional needs assessment of a scientific project (PArC‐AVE)		249	20.79 ± 4.56 [range 16–48]	13.1% male, 86.9% female	X	Focus of apprenticeship: (a) health care and nursing (*n* = 227), (b) nursing assistant (*n* = 19)
6	Patients with chronic obstructive pulmonary disease (COPD)	Baseline values of a randomized controlled trial (STAR study) in a rehabilitation clinic in Bavaria		351	58.21 ± 5.44 [range 43–85]	31.1% male, 68.9% female	X	No definition of further sub‐sample
7	Persons with lung problems (without manifest disease)	Baseline values of persons not meeting the disease inclusion criteria of a study (STAR study)	FAU Erlangen‐Nürnberg (not published)	13	54.85 ± 5.26 [range 45–63]	62.9% male, 30.8% female	X	No definition of further sub‐sample
8	Employees of different companies (in Switzerland)	Members of different exercise and sport consultation programs (baseline data) in Switzerland	University of Bern, Switzerland (Schorno et al., 2021, 2022)	644	43.54 ± 14.18 [range 18–70]	39.4% male, 60.6% female	X	Employees in the service sector: (a) health insurance company (*n* = 304), (b) hotel (*n* = 33), (c) trading company (*n* = 12), (d) bank company (*n* = 40), (e) lifting technique company (*n* = 21), (f) company 6 (*n* = 15), (g) company 7 (*n* = 57); participants of exercise/sport counseling: (h) program A (*n* = 17), (i) program B (*n* = 145)
9	Vocational students of commerce	Participants of a small student project undergoing institutional change (baseline data)	FAU Erlangen‐Nürnberg (not published)	26	19.85 ± 3.34 [range 17–30]	30.8% male, 69.2% female	X	No definition of further sub‐sample
10	Outpatient rehabilitation sports	Participants of various rehabilitation groups of an outpatient rehabilitation center (cross‐sectional data)	IST University of Applied Sciences Düsseldorf (not published)	134	62.45 ± 13.63 [range 27–97]	23.5% male, 75.8% female	X	No definition of further sub‐sample
11	Persons with Morbus Crohn	Online survey for disease‐specific associations, self‐help groups, and social media groups	IST University of Applied Sciences Düsseldorf (not published)	228	44.68 ± 13.20 [range 18–76]	27.8% male, 72.2% female	X	No definition of further sub‐sample
12	Persons with Colitis Ulcerosa	Online survey for disease‐specific associations, self‐help groups, and social media groups		173	43.24 ± 14.57 [range 19–76]	39.9% male, 60.1% female	X	No definition of further sub‐sample
13	Employees of a large industrial manufacturing company in Germany	Baseline data of a prevention program against metabolic syndrome, part of occupational health management	Technical University of Munich (Blaschke et al., 2021)	827	49.05 ± 8.05 [range 23–65]	73.7% male, 26.3% female	X	Type of work: (a) office workers (*n* = 744), (b) manufacturing (*n* = 78)
14	Participants of health care in centers of Styria, Austria	Baseline procedure of a recruitment at a community health center and through six general practitioners	University of Graz, FH Joanneum Bad Gleichenberg, Austria (Holler et al., 2023)	238	48.47 ± 16.77 [range 15–85]	38.2% male, 61.8% female	X	Institution: (a) outpatient clinic (*n* = 151), (b) health center 1 (*n* = 54), (c) health center 2 (*n* = 19), (d) incomplete information (*n* = 14)
15	Persons with multiple sclerosis (PwMS)	Cross‐sectional survey with members of a mailing list and disease‐specific social media groups	FAU Erlangen‐Nürnberg (Carl, Sudeck, & Pfeifer, 2020)	475	47.76 ± 10.03 [range 24–77]	26.5% male, 73.5% female	X	Forms of multiple sclerosis: (a) relapsing (*n* = 293), (b) primary progredient (*n* = 39), (c) secondary progredient (*n* = 110), (d) other form (*n* = 33)
16	Teaching students	Cross‐sectional survey with participants of a minor‐degree physical education certificate		502	23.14 ± 3.71 [range 18–44]	12.2% male, 87.8% female	X	Teaching students: (a) elementary school (*n* = 309); (b) middle school (*n* = 135); (c) special education (*n* = 56)
17	Cancer survivors	Cross‐sectional data with patients at the National Center for Tumor Diseases (PEXO‐M study)	University of Heidelberg (for the MM collective (Kuehl et al., 2023)	126	64.10 ± 9.50 [range 24–77]	57.1% male, 42.9% female	X	Sub‐form: (a) multiple myeloma (MM; *n* = 98); (b) smoldering multiple myeloma (SMM; *n* = 28)
18	Persons with spondyloarthritis	Baseline values of a long‐term study (routine data) at the university hospital Erlangen	FAU Erlangen‐Nürnberg (not published)	106	51.25 ± 12.61 [range 22–84]	54.4% male, 45.6% female	X	Subform: (a) psoriatic arthritis (*n* = 52); (b) axial spondyloarthritis (*n* = 51)
Total	18 independent samples (8 can be referred to the prevention sector, 10 to the rehabilitation sector)			7233/7134^b^	40.37 ± 16.78 years [range 15‐97]	39.1% male, 60.9% female	All	Analysis: *N*_subsamples_ = 48

^a^ If available.

^b^ The number before the slash (/) refers to all potential participants across the primary datasets (also including a 19th dataset with *n* = 49 car manufacturers without any information on age); the number after the slash (/) refers to the number of participants whose affiliated sub‐sample met the age assessment and power criterion (*n* ≥ 10).

^c^ The percentages refer to valid data only (missing: *n* = 178; 2.5%); there was *n* = 1 person self‐defining as non‐binary.

### 2.2 Materials

Over the past 7 years, an increasing number of studies have suggested opportunities to assess PAHCO. A three‐factor variant involved operationalizations for control of physical load and affect regulation as representing control competence and for self‐control as representing self‐regulation competence (Sudeck & Pfeifer, 2016). In a stepwise approach, this instrument has been extended by further operationalizations (also covering the model component of movement competence), finally resulting in a ten‐factor measurement (see Table 2) that combined competence facets and basic elements of the PAHCO model. Moreover, drawing on conceptual descriptions by Rheinberg and Engeser (2010), items have been developed recently for motivational competence in exercise and sport (Schorno et al., 2021).

**TABLE 2:** Linear and quadratic associations of the PAHCO indicators with age, under consideration of sample effects in the multilevel models (random intercept models).

PAHCO indicator	Attributable sub‐competence of PAHCO	*N*	Model comparison^a^	Linear models					Quadratic models			
				*b*	*t*	*p*	*β*	$R_{\text{marg}}^{2}$	*b*_linear_	*b*_quad_	*p*_prediction_	$R_{\text{marg}}^{2}$
Manageability of endurance demands (MED; 4 items)	Movement competence	2668	Δ*χ* ^2^(1) = 5.44, *p* = 0.020*	−0.058	−7.64	<0.001***	−0.225 [−0.283, −0.168]	0.068	0.026	−0.0009	<0.001***	0.054
Manageability of strength demands (MSD; 4 items)	Movement competence	2669	Δ*χ* ^2^(1) = 21.1, *p* < 0.001***	−0.104	−15.3	<0.001***	−0.400 [−0.451, −0.349]	0.251	0.043	−0.0016	<0.001***	0.211
Manageability of balance demands (MBD; 5 items)	Movement competence	2631	Δ*χ* ^2^(1) = 5.42, *p* = 0.020*	−0.146	−12.5	<0.001***	−0.341 [−0.394, −0.288]	0.128	−0.018	−0.0014	<0.001***	0.107
Body and movement awareness (5 items)	Control competence, movement competence	1918	Δ*χ* ^2^(1) = 0.774, *p* = 0.379	−0.017	−2.06	0.040*	−0.066 [−0.131, −0.003]	0.005	Not superior to linear model			
Control of physical load (6 items)	Control competence	6897	Δ*χ* ^2^(1) = 0.001, *p* = 0.973	0.009	1.39	0.165	0.028 [−0.013, 0.069]	0.003	Not superior to linear model			
Affect regulation (4 items)	Control competence	6901	Δ*χ* ^2^(1) = 0.669, *p* = 0.413	−0.008	−1.61	0.108	−0.033 [−0.073, 0.008]	0.002	Not superior to linear model			
Task‐specific self‐efficacy (3 items)	Self‐regulation competence, movement competence	2981	Δ*χ* ^2^(1) = 1.25, *p* = 0.264	−0.063	−10.1	<0.001***	−0.278 [−0.333, −0.224]	0.109	Not superior to linear model			
Self‐control (3 items)	Self‐regulation competence	6911	Δ*χ* ^2^(1) = 3.90, *p* = 0.048*	0.019	5.17	<0.001***	0.106 [0.065, 0.145]	0.015	−0.016	0.0004	<0.001***	0.012
Emotional attitude toward physical activity (4 items)	Self‐regulation competence	4001	Δ*χ* ^2^(1) = 0.200, *p* = 0.655	0.032	3.36	<0.001***	0.088 [0.036, 0.140]	0.009	Not superior to linear model			
Cognitive attitude toward physical activity (4 items)	Self‐regulation competence	2978	Δ*χ* ^2^(1) = 5.76, *p* = 0.016*	−0.004	−0.705	0.481	−0.019 [−0.072, 0.033]	0.007	0.068	−0.0008	0.044*	0.008

*Note*: All linear and quadratic models were adjusted for gender (−0.079 ≤ *β* ≤ 0.278; 1 = female, 2 = male); *β* = standardized beta coefficient.

Abbreviation: PAHCO, physical activity‐related health competence.

^a^ We compared linear versus quadratic associations between the PAHCO indicators and age, with non‐significant test statistics indicating non‐superiority of quadratic models (following the rule of parsimony, we then preferred linear models).

*p < 0.05, ***p < 0.001.

The data pooling revealed that only one project registered motivational competence at the time point of this data collection so far. Therefore, we did not consider this indicator for the present data pooling analysis. However, all studies involved the three‐factor PAHCO variant, in most cases complemented through further selected indicators in line with the project goal, sometimes the comprehensive ten‐factor variant. Further information regarding the included indicators across the primary studies, the number of items per scale, their assignment to the theoretical PAHCO sub‐components, and their reliability (via Cronbach's *α*) within this entire dataset can be retrieved from Table 2 and Supplementary Table S2. Further details of the instruments, including aspects of factorial and criterion validity, relevant for this study can be found in three methodological articles (Carl, Sudeck, Geidl, et al., 2020; Carl, Sudeck, & Pfeifer, 2020; Sudeck & Pfeifer, 2016).

We extracted participants' age (continuous variable) and gender (dichotomous variable: 1 = female, 2 = male) as common sociodemographic information from the primary studies. Unfortunately, not all scientific projects assessed an individual's health, PA level (in particular, not with the same instruments), as well as height and weight. As these variables were not essential for answering the present research questions, we did not consider these variables for this analysis.

### 2.3 Procedures and analyses

For the present study, we only included participants from the entire data pooling whose sub‐sample (a) provided an assessment on age and (b) has a size that was statistically eligible to fit with the requirements of multilevel modeling (*n* ≥ 10; McNeish & Stapleton, 2016). One sub‐sample (with *n* = 49 car manufacturers) did not assess age and nine sub‐samples (*n* = 50 participants) were too small (e.g., having a specific sub‐form of a disease or an exceptional vocational background), which led to their exclusion. A total of 7134 individuals across 48 disjunct sub‐samples met these criteria and hence, entered the analyses (see Table 1).

Individual data was nested within their primary studies covering a broad range of target groups and settings. We accounted for this clustering by applying a successive multilevel approach with the individual data as the first‐level factor and the sub‐sample as the second‐level factor. However, in the first step, we calculated random‐intercept‐only models (RIOMs) separately for each PAHCO indicator as a requirement for the consideration of the hierarchical structure of the data. In this context, intraclass correlation coefficients (ICC) were determined for the second level of the model. From an inferential statistical perspective, we compared the RIOM to a null model without any hierarchical structure (only with a fixed intercept). In the second step, we specified random‐intercept models (RIMs) by encompassing the participant‐level age data (first level) as linear (*X*) predictors to test hypothesis 1. From this point, we consistently integrated gender as a linear covariate (fixed effect) into the model. In the third step, we complemented the same RIMs through a quadratic term (*X* ^2^) allowing for an exploration of non‐linear associations across the life span (similar as to the non‐linear patterns registered between age and PA behavior (Varma et al., 2017). Subsequently, we statistically compared the linear and quadratic prediction through model comparisons. Following the principle of parsimony (Ockham's Razor; see Lazar, 2010), we only preferred the quadratic variant in case of statistical superiority. In the fourth step, we extended the linear or quadratic variants, respectively, to random‐slopes models (RSMs), in which we allowed the slope (i.e., the associations between age and PAHCO) to fluctuate randomly across the sub‐samples (group level). In direct model comparisons, we examined in line with hypothesis 2 whether the dataset favored statistical models with an invariant or a random slope (across the defined sub‐samples).

We compared all models with restricted maximum likelihood estimators. We outlined the linear variants for all PAHCO indicators by both unstandardized (*b*) and standardized (*β*) beta coefficients. The degrees of freedom for the predictors were estimated with the Kenward Roger correction to extract information about the statistical significance of variables in the multilevel models, as this procedure enables robust estimations also with small sample sizes per cluster (McNeish, 2017). Nevertheless, we followed the suggestion to only include clusters (i.e., sub‐samples) with at least 10 individuals (McNeish & Stapleton, 2016). We performed the Chi‐squared (*χ* ^2^) test (via −2ΔLL likelihood ratio test) for all statistical model comparisons (null model vs. RIOM; linear vs. quadratic association; invariant vs. random slopes). The explained variance of age and gender as predictors within the multilevel models were inspected by examining the marginal determination coefficient ($R_{\text{marg}}^{2}$) for fixed effects (Nakagawa & Schielzeth, 2013). We used expectation maximization‐based imputation procedures (Lüdtke et al., 2007) to counteract missing values for PAHCO, but only if one item was missing for each scale. If more information was missing per indicator, the corresponding scale was treated as missing and did not contribute to the model for this person. We localized the significance level at *p* < 0.05, implying that the pooled dataset had the potential to detect effects *β* ≥ 0.064 for the PAHCO variable with the smallest (*n* = 1918) and effects *β* ≥ 0.034 for the variable with the largest sample size (*n* = 6911). We ran all statistical analyses with the software R (version 4.1.3) and the lmerTest package (Kuznetsova et al., 2017). The presentation of the associations was complemented through graphs (linear or quadratic) with the predicted mean curve as continuous lines and the corresponding confidence intervals as dashed lines. The confidence intervals were computed manually with Excel 2018 by drawing on the following formula:

(1) $$y_{ci}={\hat{y}}_{k}\pm \;t_{\alpha /2,n-2}\cdot \sqrt{MSE\cdot \left(\frac{1}{n}+\frac{{\left(x_{k}-\;\bar{x}\right)}^{2}}{\sum {\left(x_{i}-\bar{x}\right)}^{2}}\right)}\;\left(\text{MSE}=\text{mean}\;\text{standard}\;\text{error}\right)$$

## 3 RESULTS

### 3.1 Requirements of multilevel analyses

All PAHCO indicators were nested in their sub‐sample (0.031 ≤ *ICC* ≤ 0.365), with the manageability of balance demands (MBD) indicator showing the highest and cognitive attitude toward PA as the lowest coefficient (Supplementary Table S2). Also from an inferential statistical perspective, models considering the sub‐sample as second‐order factor fitted significantly better than simple models with only the PAHCO indicators as predictors (29.5 ≤ Δ*χ* ^2^(1) ≤ 973, *p* < 0.001). Therefore, multilevel modeling was indicated for all 10 variables under investigation.

### 3.2 Associations with age

Four PAHCO indicators could be expressed by linear regression models across age, while their extension to quadratic prediction models did not significantly improve their model fit (Table 2): body and movement awareness, control of physical load, affect regulation, task‐specific self‐efficacy, and emotional attitude toward PA. In contrast, the associations of five PAHCO indicators with age could be better represented by second‐degree polynomial regressions: manageability of endurance demands (MED), manageability of strength demands (MSD), MBD, self‐control, and cognitive attitude toward PA.

Table 2 illustrates the relationships with age separately for each PAHCO indicator within the RIMs. MED ($R_{\text{marg}}^{2}$ = 0.054, *p* < 0.001), MSD ($R_{\text{marg}}^{2}$ = 0.211, *p* < 0.001) and MBD ($R_{\text{marg}}^{2}$ = 0.107, *p* < 0.001) displayed reductions of increasing intensity across the life span (moderate effect size). The curve of body and movement awareness (*β* = −0.066, *t* = −2.06, *p* = 0.040) also decreased significantly with a small effect size. Control of physical load (*β* = 0.028, *t* = 1.39, *p* = 0.165) and affect regulation (*β* = −0.033, *t* = −1.61, *p* = 0.108) were not significantly related to chronological age, thus remaining relatively stable throughout the modeled life course. Task‐specific self‐efficacy declined moderately with increasing age (*β* = −0.278, *t* = −10.1, *p* < 0.001), whereas self‐control ($R_{\text{marg}}^{2}$ = 0.012, *p* < 0.001) and emotional attitude toward PA (*β* = 0.088, *t* = 3.36, *p* < 0.001) improved throughout the chronological trajectory (small effect). Finally, cognitive attitude toward PA can be best described by an inverted u‐shaped association with age ($R_{\text{marg}}^{2}$ = 0.008, *p* = 0.044), finding its peak at the age of 44 (small variance explained).

**FIGURE 2:**
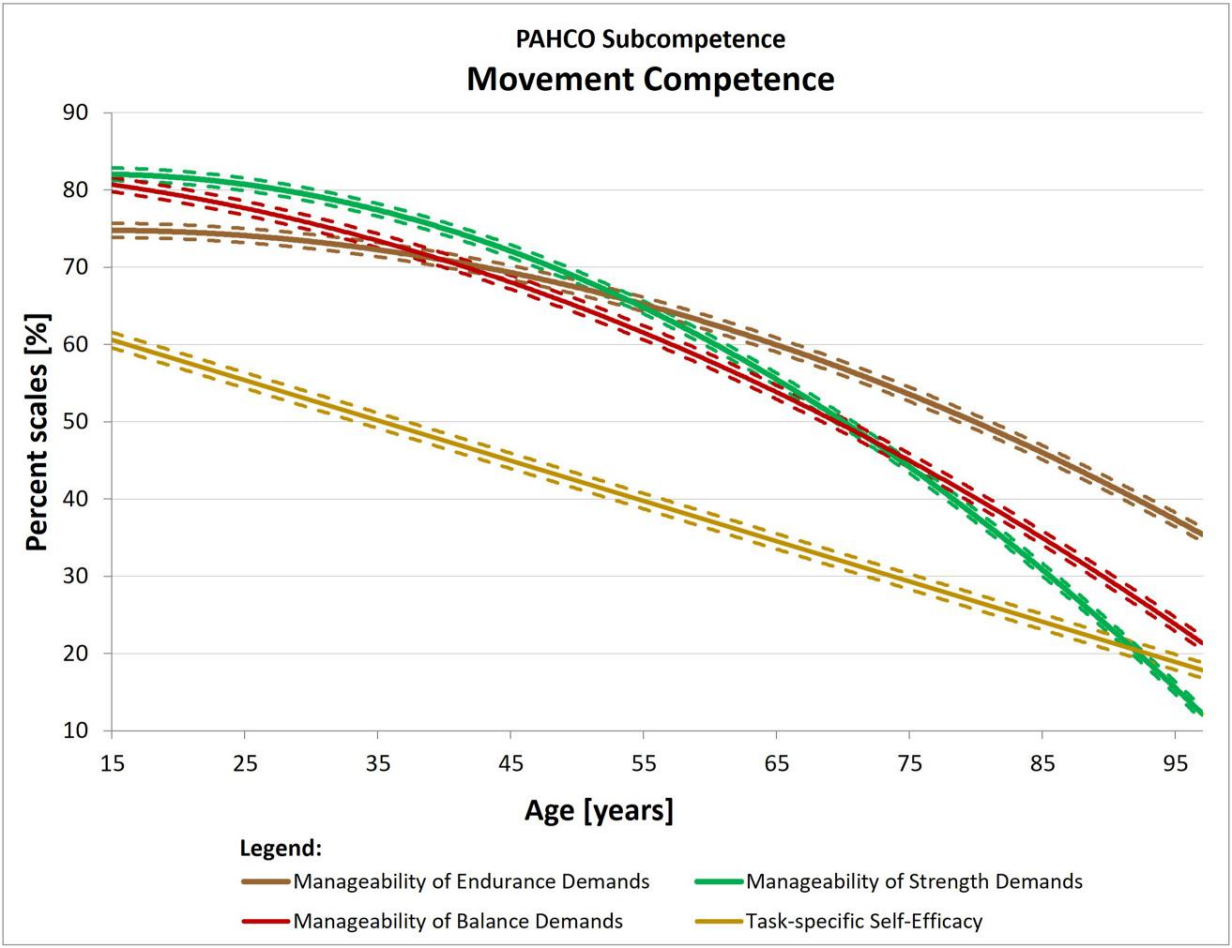
The modeled courses of the movement competence indicators across the life span. The solid line marked the modeled mean curve, while the dotted lines around the mean curve represented the 95% confidence intervals for the mean curve. For plotting all curves into one chart, we have transformed all values to percent scales.

**FIGURE 3:**
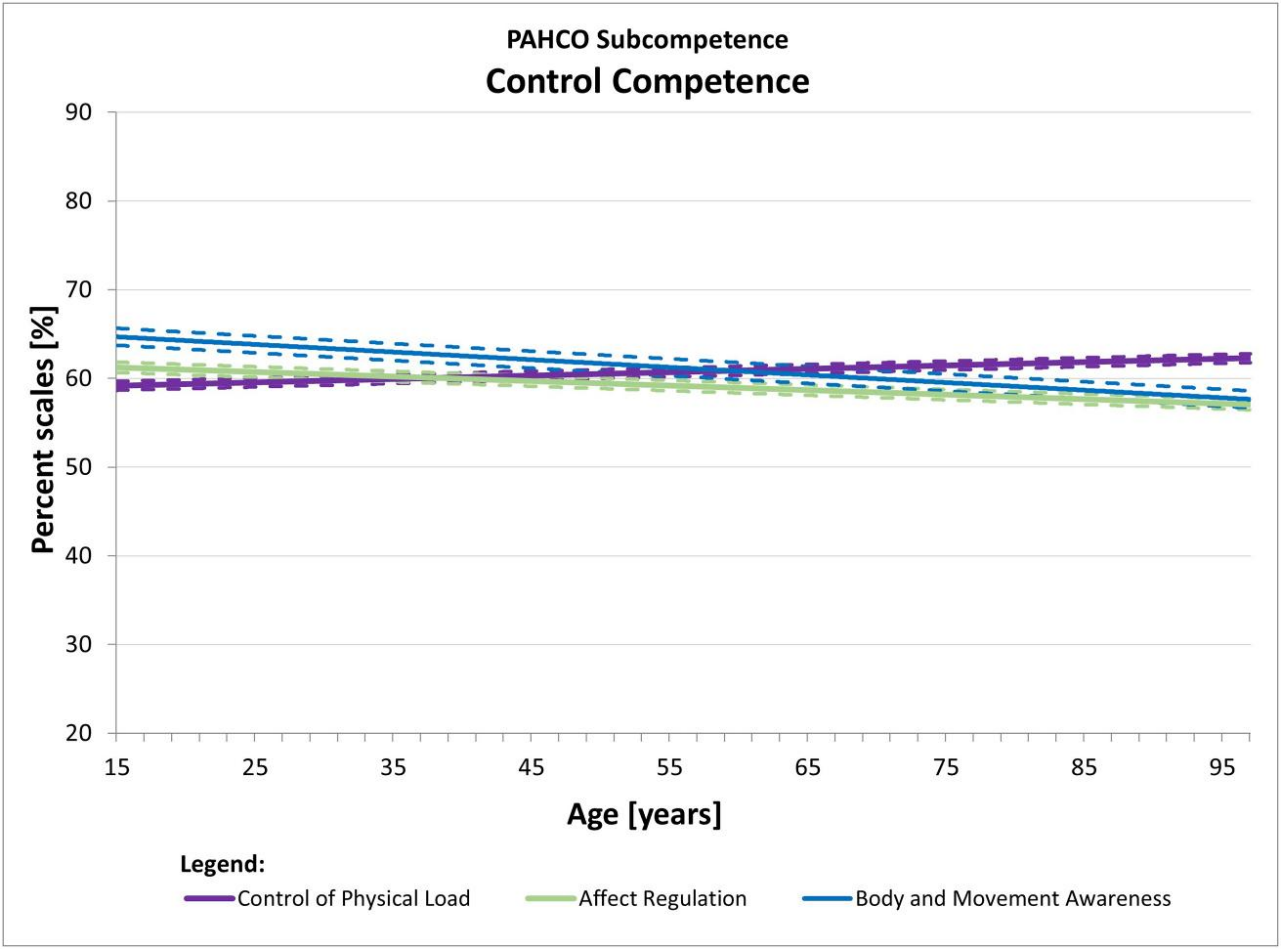
The modeled courses of the control competence indicators across the life span. The solid line marked the modeled mean curve, while the dotted lines around the mean curve represented the 95% confidence intervals for the mean curve. For plotting all curves into one chart, we have transformed all values to percent scales.

**FIGURE 4:**
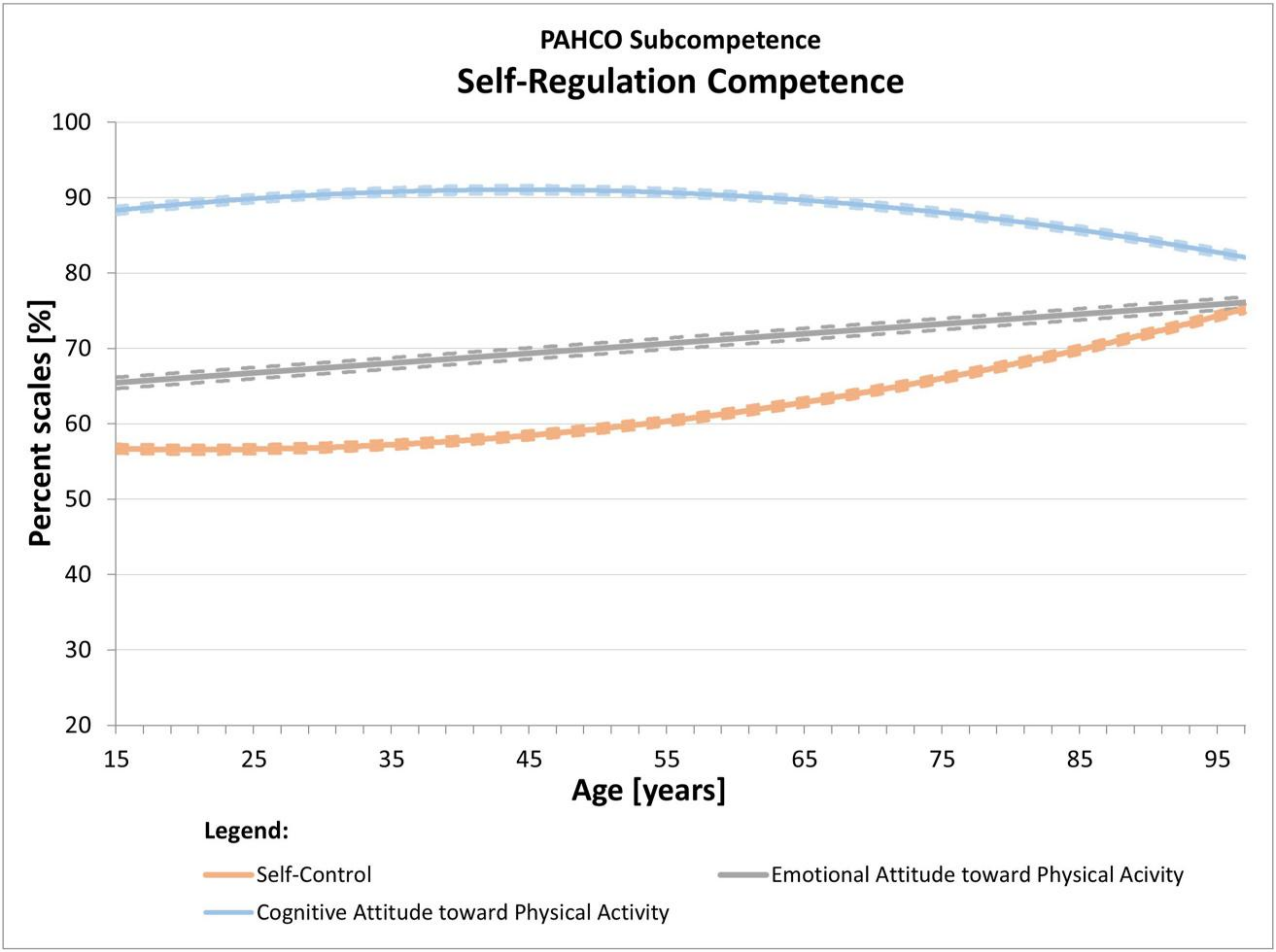
The modeled courses of the self‐regulation competence indicators across the life span. The solid line marked the modeled mean curve, while the dotted lines around the mean curve represented the 95% confidence intervals for the mean curve. For plotting all curves into one chart, we have transformed all values to percent scales.

If the effects are globally interpreted by assigning the 10 indicators to the sub‐competences of the PAHCO model in accordance with the highest theory‐compatible loadings found in a second‐order analysis (Carl, Sudeck, & Pfeifer, 2020), we detected the following pattern: the modeled levels of all main indicators of *movement competence* (MED, MSD, MBD, task‐specific self‐efficacy) consistently declined with age (Figure 2). The main indicators of *control competence* (control of physical load, affect regulation), in turn, revealed no significant associations with age (Figure 3). Only the course of body and movement awareness—theoretically and empirically located at the interface to movement competence—slightly decreased across the life span. Finally, the indicators self‐control and emotional attitude toward PA—conceptually attributable to *self‐regulation competence*—significantly increased throughout the life course, while the quadratic relationship of cognitive attitude toward PA pointed to potential reductions in later life (Figure 4).

### 3.3 Differential associations across target groups

We maintained assumptions of sample‐invariant association across age for the PAHCO indicators of body and movement awareness, control of physical load, task‐specific self‐efficacy, emotional attitude toward PA, as well as cognitive attitude toward PA (Supplementary Table S3). For these model indicators, the empirical data favored the representation of a single slope across age. However, the statistical comparison of model fits suggested that the associations of MED (Δ*χ* ^2^(5) = 17.4, *p* = 0.004), MBD (Δ*χ* ^2^(5) = 31.5, *p* < 0.001), MSD (Δ*χ* ^2^(5) = 21.1, *p* < 0.001), self‐control (Δ*χ* ^2^(5) = 11.3, *p* = 0.045), and affect regulation (Δ*χ* ^2^(2) = 7.53, *p* = 0.023) with age varied significantly across the sub‐samples (RSM).

## 4 DISCUSSION

Drawing on the PAHCO model (Carl, Sudeck, & Pfeifer, 2020; Sudeck & Pfeifer, 2016) and on multilevel modeling with a large dataset pooling initiative, the present study ascertained that the direct movement‐related requirements may consistently diminish with age (movement competence), while the ability to align PA with an individual's holistic health remained stable across the entire adulthood (control competence) and the motivational indicators for ensuring the regularity of PA may even slightly improve with age (self‐regulation competence). Moreover, the analyses revealed that the associations of age with some PAHCO indicators (MED, MSD, self‐control, affect regulation) appear to vary between target groups.

The empirical findings corroborated our hypothesis that movement competence significantly changed across the chronological trajectory. Indeed, previous studies analyzing single aspects of movement competence already pointed toward this direction. For instance, two studies found reductions in interoceptive accuracy and awareness with age (Khalsa et al., 2009; Murphy et al., 2018). Similarly, chronological age was also negatively associated with physical fitness (Kimura et al., 2012; Tveter et al., 2014) as well as with the ability to perform instrumental activities of daily living (Liao & Chang, 2020; Mueller‐Schotte et al., 2020). In this regard, it can be interpreted as a hint for the content validity of the self‐reported PAHCO assessment that movement competence, as an aggregate concept bundling different physical aspects and qualities, consistently showed these associations. Interestingly, these relationships not only refer to the capability to master external—basically objectifiable—strength, endurance, and balance demands but also to the (subjective) feeling of being able to perform challenging physical activities (self‐efficacy).

Adopting an exploratory approach, we did not specify any hypotheses for the indicators of control competence. Despite registering a slight decrease in body and movement awareness with higher age, our findings demonstrated that individuals can appropriately align their physical activities toward health across the entire adulthood, even in the elderly. From the perspective of health‐enhancing PA, practitioners are encouraged to maintain quality of supervision and consultation irrespective of an individual's age. Studies have underlined that therapists, coaches, and consultants tend to treat individuals differently (ageism) following the mere exposure of age characteristics (Jin & Harvey, 2020; Swift et al., 2017). In this regard, the present findings strongly question potential prejudices by professionals that PAs might not substantially contribute to the health of elderly people. If professionals truly internalized a person‐centered approach, they should take corresponding resources to improve this alignment process. Specific to the PAHCO model differentiating between control competence for physical health (“control of physical load”) and psychological health (“affect regulation”), this could mean that professionals attempt to identify optimal physical loads in interaction with the target person (Coyne et al., 2018; Garber et al., 2011; Thiel et al., 2018) and to improve the fit with individuals' preferences and motive constellations (Ekkekakis, 2009; Schorno et al., 2022; Sheldon & Elliot, 1999; Sudeck et al., 2018). In this context, future insights regarding motivational competence—within the scope of this dataset pooling, only one study has included this indicator—could enrich operationalizations and applications of PAHCO. In terms of self‐regulation competence, we even identified positive developments over the life span. One reason for the increases of emotional attitudes toward PA could be that individuals in older age may have more favorable associations with PA, as they, in accordance with the socioemotional selection hypothesis (Carstensen, 2021), tend to prefer social contexts when executing exercises (Steltenpohl et al., 2019). Furthermore, older persons reported increasing self‐control for implementing PA, which may be explained by an increasing relevance and prioritization of health aspects with age or fewer temporal barriers given the receding occupational involvement. Finally, the inverted u‐shaped course of cognitive attitudes toward PA may result from successively positive health persuasions regarding the benefits of PA until the middle of the forties but afterward also with the growing self‐awareness that an active lifestyle can only partially mitigate the accelerating aging processes occurring in later stages of life.

Taken together, the PAHCO curves and their distinction into sub‐competence and indicators underlined the value of a differentiated approach to analyzing individual requirements for health‐enhancing PA. The findings have the potential to complement existing studies on PA levels demonstrating a decline around the age of 50 years, especially among men (Varma et al., 2017). When comparing the curves of ontogenetic PA curves with those of the present study, we identified the strongest parallels with the domain of movement competence. However, one advantage of the PAHCO model is that the PA behavior surpasses the quantitative level by also incorporating a qualitative dimension, and that the adopted educational notion of “competence” has led into tangible assumptions on how to arrange practices for health‐enhancing PA. According to corresponding action models (Carl, Sudeck, & Pfeifer, 2020; Gleddie & Morgan, 2020; Sudeck & Pfeifer, 2016), interventions should simultaneously consider and intertwine exercise/training (physical orientation), learning (cognitive orientation), and experiences (affective orientation) with behavior. Specific to this study and to practices with aging relevance, stakeholders should deliver the important message that individuals can achieve health goals and gains by an active lifestyle throughout the life course, with realistically larger subjective degrees of freedom for regular embedment into daily life. In accordance with an explicit approach to (cognitive) learning, we recommend to install a non‐directive atmosphere for health professionals' conversations with clients or patients to access “reflection in action” or “reflection on action” as favored by PAHCO (Carl, Sudeck, & Pfeifer, 2020; O’Halloran et al., 2014). When working with PA novices, therapists, coaches, and consultants can draw on biographical methods to multi‐dimensionally “chart” an individual's journey of physical (in)activity for a better localization of oneself within the familiarization process toward a (more) physically active lifestyle (Green et al., 2018; Mayer et al., 2020; Schubring et al., 2019). Such a procedure makes subjective trajectories, which could only be modeled cross‐sectionally in this study, on an individual level visible. As health knowledge and health care is often delivered via professionals (i.e., therapists, coaches, or educators), these findings should also reach these mediators to nourish a de‐stigmatizing approach to better exhaust the health potential in the elderly (Swift et al., 2017). Accordingly, we encourage education and training on PA to include content on PAHCO across the life span.

The present study had the following limitations. First, the findings regarding the associations between age and PAHCO relied on cross‐sectional data. Even though an inverse explanation outlining an effect of PAHCO on chronological age can be logically excluded, this methodological constellation (without further confounding variables beyond gender) weakened causal inferences. Second, some particularities of the included populations—despite their covered great variety across the prevention and rehabilitation settings—may have affected the pattern across the life span. For instance, the dataset incorporated several diseases (e.g., multiple sclerosis or cancer) which bring into play additional challenges for the regulation of PA. At this point, we attempted to counteract this situation by considering data nesting through the application of multilevel modeling. Relatedly, very old individuals (≥75 years) were slightly underrepresented in the dataset. Third, we defined the subsamples in dependency of the primary studies, which implies—irrespective of their disjunct character—that their formation based on different criteria, hierarchical levels, and number of included persons. Fourth, the primary studies drew on different versions of PAHCO, depending on the advancement and availability at the respective stages. The included empirical studies used a mixture of operationalizations on the levels of competence and basic elements. Stronger consistency may have enhanced the power for some PAHCO indicators and positively affected robust conclusions. Fifth, the PAHCO indicators could not be linked to PA levels in the present analysis. This study would have been conceptually and empirically enriched by bridging the gap between competence and behavior, if all primary studies had adopted a similar operationalization of PA. Sixth, although we controlled for gender, further socioeconomic conditions (e.g., education or income) play a role for health‐enhancing PA and their adjustment would have strengthened the implications from the associative patterns. Lastly, all studies came from the German‐speaking area. In this regard, previous research on PAHCO has not yet exhausted its potential in terms of cross‐cultural generalizability.

In recent years, literature has increasingly discussed the relevance of competencies and literacy as requirements for active lifestyles (Buja et al., 2020; Cairney et al., 2019; Sudeck et al., 2022; World Health Organization, 2018). However, as there have been scant insights regarding the development of these personal factors across the life span, the strength of this study was that it empirically drew on pooled primary datasets combining a variety of populations across the life span. While researchers have generated insights into mean curves for PA levels, this endeavor left the behavioral level and outlined multifaceted competencies for healthy, physically active lifestyles throughout the life course. To the best of our knowledge, no study has yet simultaneously modeled physical, cognitive, and motivational requirements for health‐enhancing PA across such an age spectrum. In this regard, the PAHCO model has delivered a clear framework underlying the present analyses.

## 5 CONCLUSION

Comprehending the course of competencies for healthy, active lifestyles is paramount to improve and optimize health‐enhancing PA, irrespective of whether these are undertaken during instrumental activities of daily living, leisure time, and planned therapeutic settings. As expected, the results suggest age‐related declines in movement competence, which stresses the importance of high‐quality training concepts to avoid unnecessary functional restrictions and delay or compress morbidity in the elderly. Researchers have accumulated many insights pointing to the plasticity of movement competence by well‐designed interventions throughout the entire life course. In accordance with a holistic consideration of PA, the present study uncovered an unaffected ability to align PA with health across the life course and even a promising reserve to ensure regularity of PA. In this regard, practitioners should be aware of this potential and, counterintuitive to aging stereotypes, invest the deserved efforts in closely following the developments of older individuals, especially if health is conceptualized in a multidimensional, biopsychosocial way. However, as the modeled courses represented aggregated data across individuals and different populations, practitioners are, for high‐quality care, advised to exactly know an individual's PAHCO situation and translate a unique person profile (e.g., based on a typology approach) into adequate intervention delivery.

## AUTHOR CONTRIBUTIONS

*Conceptualization*: Johannes Carl. *Methodology*: Johannes Carl and Simon Blaschke. *Software*: Johannes Carl, Simon Blaschke, and Johannes Jaunig. *Validation*: Simon Blaschke. *Formal analysis*: Johannes Carl. *Investigation (primary studies)*: all authors. *Resources (primary studies)*: all authors. *Data Curation*: Johannes Carl. *Writing—original draft*: Johannes Carl. *Writing—review and editing*: all authors. *Visualization*: Johannes Carl. *Supervision*: Johannes Carl, Klaus Pfeifer, and Gorden Sudeck. *Project administration*: Johannes Carl. *Funding* *acquisition (primary studies)*: see primary studies. *Funding* *acquisition (data pooling)*: not applicable.

## CONFLICT OF INTEREST STATEMENT

The authors report there are no competing interests to declare.

## Supporting information

Supporting Information S1

## Data Availability

All responsible researchers with their ownership regarding the primary datasets provided informed consent to data pooling.
