# Supplementary material for: A life span perspective on competencies for a healthy, physically active lifestyle: Findings of a data pooling initiative with over 7000 individuals
Source: Eur J Sport Sci. 2024 Apr 16;24(6):788–803. doi: 10.1002/ejsc.12100 (PMC11236042; doi:10.1002/ejsc.12100)
Supplement: Supplementary file 1 — Supporting Information S1 [file EJSC-24-788-s001.docx]

**Supplementary Tables**

Supplementary Table 1. *More detailed information about inverting and recoding processes.*

| **Type** | **Reason and Transformation** | **Concerned Samples** | **Previous values** | **Values after** |
| --- | --- | --- | --- | --- |
| Recoding a scale | Transformation of a four-point Likert scale (without response locations for the medium options) into a five-point Likert scale (also without response locations for the medium options) | 1, 13, 14, 17 | Three scales (control of physical load, affect regulation, self/control): 1 – does not apply 2 –  3 –  4 – applies very much | Three scales (control of physical load, affect regulation, self/control): 1 – does not apply 2.33 -  3.67 -  5 – applies very much |
| Recoding a scale | Transformation of a 0-4 scale to an inverted 1-5 scale | 11, 12 | Manageability or endurance demands and manageability of strength demands scales: 0 – I cannot do this 1 – I have major problems 2 – I have moderate problems 3 – I have some slight problem 4 – I can do this without any problems | Manageability or endurance demands and manageability of strength demands scales: 1 – I can do this without any problems 2 – I have some slight problem 3 – I have moderate problems 4 – I have major problems  5 – I cannot do this |
| Inverting | Gender | 3, 4, 5, 6, 7, 9, 14, 15, 16 | 1 – male  2 – female  (3 – other) | 1 – female  2 – male  (3 – other) |

Supplementary Table 2. *Examination of the sub-sample effect and the type of association.*

| **PAHCO Indicator** | **Number of Items** | **Attributable sub-competence of PAHCO** | **Assessed in  Studies^1^** | ***α*** | ***N*** | **Sample/Group Relevance** | | **Evaluating the Type (Linear vs. Quadratic) of Association^3^** | |
| --- | --- | --- | --- | --- | --- | --- | --- | --- | --- |
|  |  |  |  |  |  | ***ICC*** | **Comparison with non-hierarchical model^2^** | **Test Statistics of Model Comparison** | **Decision** |
| Manageability of endurance demands (MED) | 4 | Movement competence | 3, 4, 5, 9, 10, 11, 12, 14, 15, 16, 18 | 0.912 | 2668 | 0.236 | Δ*χ*²(1) = 480,  *p* < .001*** | Δ*χ*²(1) = 5.44,  *p* = .020* | Quadratic model |
| Manageability of strength demands (MSD) | 4 | Movement competence | 3, 4, 5, 9, 10, 11, 12, 14, 15, 16, 18 | 0.915 | 2669 | 0.303 | Δ*χ*²(1) = 756,  *p* < .001*** | Δ*χ*²(1) = 21.1,  *p* < .001*** | Quadratic model |
| Manageability of balance demands (MBD) | 6 | Movement competence | 3, 4, 5, 9, 10, 11, 12, 14, 15, 16, 18 | 0.951 | 2631 | 0.365 | Δ*χ*²(1) = 973,  *p* < .001*** | Δ*χ*²(1) = 5.42,  *p* = .020* | Quadratic model |
| Body and movement awareness | 5 | Control competence, movement competence | 3, 9, 10, 11, 12, 14, 15, 16, 18 | 0.882 | 1918 | 0.046 | Δ*χ*²(1) = 42.6,  *p* < .001*** | Δ*χ*²(1) = 0.774,  *p* = .379 | Linear model |
| Control of physical load | 6 | Control competence | All studies | 0.860 | 6897 | 0.077 | Δ*χ*²(1) = 562,  *p* < .001*** | Δ*χ*²(1) = 0.001,  *p* = .973 | Linear model |
| Affect regulation | 4 | Control competence | All studies | 0.894 | 6901 | 0.099 | Δ*χ*²(1) = 747,  *p* < .001*** | Δ*χ*²(1) = 0.669,  *p* = .413 | Linear model |
| Task-specific self-efficacy | 3 | Self-regulation competence, movement competence | 3, 4, 5, 6, 7, 9, 10, 11, 12, 14, 15, 16, 18 | 0.896 | 2981 | 0.184 | Δ*χ*²(1) = 557,  *p* < .001*** | Δ*χ*²(1) = 1.25,  *p* = .264 | Linear model |
| Self-control | 3 | Self-regulation competence | All studies | 0.860 | 6911 | 0.055 | Δ*χ*²(1) = 254,  *p* < .001*** | Δ*χ*²(1) = 3.90,  *p* = .048* | Quadratic model |
| Emotional attitude toward physical activity | 4 | Self-regulation competence | 2, 3, 4, 5, 6, 7, 9, 10, 11, 12, 14, 15, 16, 18 | 0.959 | 4001 | 0.085 | Δ*χ*²(1) = 346,  *p* < .001*** | Δ*χ*²(1) = 0.200,  *p* = .655 | Linear model |
| Cognitive attitude toward physical activity | 4 | Self-regulation competence | 2, 3, 9, 10, 11, 12, 14, 15, 16, 18 | 0.913 | 2978 | 0.031 | Δ*χ*²(1) = 29.5,  *p* < .001*** | Δ*χ*²(1) = 5.76,  *p* = .016* | Quadratic model |

Notes and abbreviations: All linear and quadratic models were adjusted for gender (-.016 ≤ *β* ≤ .277; 1 = female, 2 = male); ICC = Intraclass correlation coefficient; **p* < .05, ***p* < .01, ****p* < .001.
^1^For the numerical designation of the studies, see the first column of Table 1.
^2^On an inferential statistical basis, we compared the multilevel model with the sub-sample as the second-level factor (random-effects model) with a linear model without consideration of the group structure.
^3^We compared linear vs. quadratic associations between the PAHCO indicators and age, with non-significant test statistics indicating non-superiority of quadratic models (following the rule of parsimony, we then preferred linear models).

Supplementary Table 3. *Investigating the role of sub-samples in the association patterns between PAHCO and age*.

| **PAHCO Indicator** | **Attributable sub-competence of PAHCO** | ***N*** | **Evaluating the Assumption of Slopes (Invariant vs. Random)^1^** | | |
| --- | --- | --- | --- | --- | --- |
|  |  |  | **Regression Type** | **Test Statistics of Model Comparison** | **Decision** |
| Manageability of endurance demands (MED) | Movement competence | 2668 | Quadratic model | Δ*χ*²(5) = 17.4, *p* = .004** | Random slopes |
| Manageability of strength demands (MSD) | Movement competence | 2669 | Quadratic model | Δ*χ*²(5) = 21.1, *p* < .001*** | Random slopes |
| Manageability of balance demands (MBD) | Movement competence | 2631 | Quadratic model | Δ*χ*²(5) = 31.5, *p* < .001*** | Random slopes |
| Body and movement awareness | Control competence, movement competence | 1918 | Linear model | Δ*χ*²(2) = 0.338, *p* = .844 | Invariant slopes |
| Control of physical load | Control competence | 6897 | Linear model | Δ*χ*²(2) = 2.96, *p* = .228 | Invariant slopes |
| Affect regulation | Control competence | 6901 | Linear model | Δ*χ*²(2) = 7.53, *p* = .023* | Random slopes |
| Task-specific self-efficacy | Self-regulation competence, movement competence | 2981 | Linear model | Δ*χ*²(2) = 0.012, *p* = .994 | Invariant slopes |
| Self-control | Self-regulation competence | 6911 | Quadratic model | Δ*χ*²(5) = 11.3, *p* = .045* | Random slopes |
| Emotional attitude toward physical activity | Self-regulation competence | 4001 | Linear model | Δ*χ*²(2) = 0.498, *p* = .780 | Invariant slopes |
| Cognitive attitude toward physical activity | Self-regulation competence | 2978 | Quadratic model | Better fit of model with  invariant slopes | Invariant slopes |

Notes: Comparisons between random-slope-models and random-intercept-models.
^1^The hypothesis is tested whether the associations between PAHCO and age are consistent across the included sub-samples; significant values reject this hypothesis and indicate that models fit better when these associations/slopes vary randomly between groups. All models were adjusted for gender. **p* < .05, ***p* < .01, ****p* < .001.
